# Hesperidin and Hesperetin from Orange Peel Water Extract Protect against NaIO_3_‑Induced Oxidative Damage in Retinal Pigment Epithelial Cells by Modulating PI3K/Akt/HIF-1α/BNIP3 Signaling

**DOI:** 10.1021/acs.jafc.5c03766

**Published:** 2025-10-13

**Authors:** Jui-Hsuan Yeh, Yuan-Yen Chang, Chen-Ju Chuang, Tzu-Chun Chen, Shang-Chun Tsou, Chieh-Jung Huang, Yi-Hsien Hsieh, Inga Wang, Ming-Chung Lee, Hui-Wen Lin

**Affiliations:** † Institute of Medicine, 63267 Chung Shan Medical University, Taichung 40246, Taiwan; ‡ Department of Microbiology and Immunology, School of Medicine, Chung Shan Medical University and Clinical Laboratory, Chung Shan Medical University Hospital, Taichung 402, Taiwan; § Emergency Department, 38031 St. Martin De Porres Hospital, Chiayi 600044, Taiwan; ∥ Department of Medical Research, Chung Shan Medical University Hospital, Taichung 40201, Taiwan; ⊥ Rehabilitation Sciences & Technology, 144114 University of Wisconsin-Milwaukee, Milwaukee, Wisconsin 53201-0413, United States; # Brion Research Institute of Taiwan, New Taipei 23143, Taiwan

**Keywords:** age-related macular degeneration (AMD), orange peel water extract (OPWE), sodium iodate (NaIO_3_), reactive oxygen species (ROS), apoptosis

## Abstract

Age-related macular degeneration (AMD) is a leading cause of blindness in the elderly, with oxidative stress a major causative factor. Orange peel, rich in polyphenols and flavonoids, possesses potent antioxidant and anti-inflammatory activities. This study evaluated the protective effects of an ultrasound-assisted aqueous extract (OPWE) of orange peel and its major components, hesperidin and hesperetin, against sodium iodate (NaIO_3_)-induced retinal damage. Component identification was performed using 3D-HPLC and LC/MS, and the antioxidant capacity was determined using the DPPH and ABTS assays. *In vitro*, OPWE reduced oxidative stress, mitochondrial dysfunction, and apoptosis in NaIO_3_-treated ARPE-19 cells through the PI3K/Akt and HIF-1α/BNIP3 pathways. Notably, hesperetin exhibited comparable protective effects to OPWE, restoring cell viability and inhibiting ROS production. *In vivo*, oral administration of OPWE maintained retinal morphology and function in mice induced by NaIO_3_. These findings suggest that OPWE, especially hesperetin, is a promising natural candidate for preventing oxidative stress-related retinal degeneration and maintaining retinal health.

## Graphical abstract

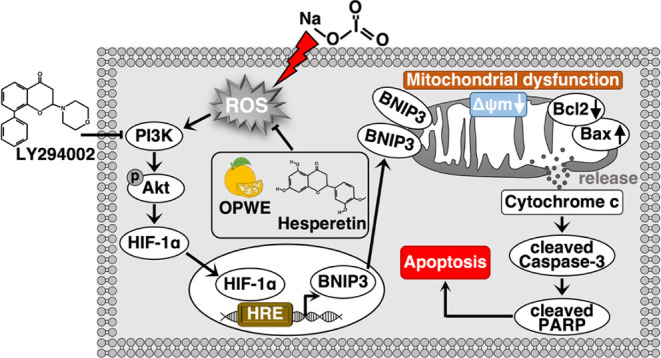

## 1 Introduction

One of the main causes of irreversible sightlessness among the elderly worldwide is age-related macular degeneration (AMD). With global population aging, AMD is projected to affect approximately 288 million individuals by 2040, posing a substantial public health burden. AMD is among the top three causes of blindness, accounting for nearly 9% of all blindness cases globally.^1,2^ AMD affects retinal pigment epithelial (RPE) cells, which maintain the blood-retinal barrier (BRB) and regulate retinal physiological functions. Excessive production of reactive oxygen species (ROS), a symbol of oxidative stress, disrupts endogenous antioxidant defense mechanisms, leading to mitochondrial dysfunction, mitochondria-dependent apoptotic pathway activation, and RPE cell death.^3,4^ Sodium iodate (NaIO_3_), an oxidizing agent, exhibits selective toxicity toward the neural retina and RPE cells and is widely used to induce AMD-like retinal damage in experimental models.^5,6^

As a core regulator of mammalian cell apoptosis, the phosphoinositide 3-kinase (PI3K)/Akt pathway has become a research hotspot due to its broad implications on various disease processes.^7–9^ In experimental models of NaIO_3_-induced retinal damage, activation of the PI3K/Akt pathway triggers downstream effectors that regulate antioxidant enzyme activity and mitochondrial function, accelerating RPE cell death and AMD progression.^10–12^ Emerging evidence highlights the pivotal role of this pathway in AMD pathogenesis, where it governs key cellular processes including proliferation, metabolism, and apoptosis.^13,14^ Notably, PI3K/Akt activation represents a compensatory response to oxidative stress, exerting a dual part in regulating cell survival and apoptosis.^15,16^

Hypoxia-inducible factors (HIFs), which function as cellular oxygen sensors, promote adaptation to hypoxic conditions by regulating oxygen-dependent genes. They also play a role in oxidative stress responses.^17,18^ HIF-1α—a key transcription factor involved in cellular responses to hypoxia—induces the expression of vascular endothelial growth factor under hypoxic conditions, increasing vascular permeability and retinal neovascularization.^19,20^ In neovascular AMD, oxidative stress triggers HIF-1α overexpression, subsequently enhancing the expression of Bcl-2/adenovirus E1B 19-kDa interacting protein 3 (BNIP3), a proapoptotic mediator that facilitates mitochondrial impairment and promotes cell death.^17,21,22^

The global production of sweet orange (*Citrus sinensis* Osbeck) exceeds 68 million tons. The peel constitutes 50%–60% of an orange’s total weight, posing environmental concerns if not managed appropriately.^23–25^ Converting peels into value-added products helps mitigate their environmental impact. Evidence suggests that sweet orange peels possess strong antioxidant, antibacterial, and antiviral properties.^26–28^ Thus, this seemingly waste product holds promise for pharmaceutical and nutraceutical applications, such as in the preparation of innovative eye health supplements and the prevention of aging-related diseases.

Citrus peels are a notable source of phenolic compounds such as phenolic acids, flavonoids, and polymethoxyflavones, which have been explored for potential health benefits.^29–31^ The peels contain abundant polyphenols, particularly flavanone glycosides such as eriocitrin, narirutin, and hesperidin. Among these compounds, hesperidin—the predominant flavonoid in orange peels-exhibits potent antioxidant properties and can cross the blood–brain barrier (BBB). Thus, hesperidin has emerged as a therapeutic candidate for mitigating oxidative stress in neurodegenerative conditions, including AMD.^32^ After oral administration of hesperidin, intestinal enzymes metabolize it into hesperetin, enhancing its bioavailability and therapeutic potential.^33,34^

This investigation aimed to determine the protective properties of orange peel water extract (OPWE) and its active components, hesperidin and hesperetin, against NaIO_3_-induced retinal oxidative damage in cellular and mouse models. It focused on oxidative stress-induced apoptosis and the HIF-1α/BNIP3 and PI3K/Akt pathways. Our findings may guide sustainable AMD management by leveraging agricultural waste.

## 2 Materials and Methods

### 2.1 OPWE Extraction

Mature oranges were collected from Gukeng, Yunlin, Taiwan, during the harvest season (November to February). After washing, the oranges were separated into pulp and peels. The peels were cut into 1 cm^2^ pieces and sterilized in distilled water at 95 °C for 1 min (peel-to-water ratio: 1:10 [g/mL]). The pieces were then cooled to 50 °C with cold water and sonicated in an ultrasonic bath for 1 h. The resultant extract was filtered into clean, sterile serum bottles, freeze-dried, and stored at −80 °C for later use. The method was adapted from Abd El-Aziz et al.^35^

### 2.2 Chemicals

The following chemicals: 2,2′-azino-bis (3-ethylbenzothiazoline-6-sulfonic acid) diammonium salt (ABTS), Folin–Ciocalteu reagent, and H_2_O_2_ were procured from Merck (Darmstadt, Germany). Na_2_CO_3_, NaNO_2_, AlCl_3_, and NaOH were obtained from Daejung Chemicals & Metals (Gyeonggi-do, Korea). Peroxidase, hesperidin, and hesperetin were purchased from Santa Cruz Biotechnology (Dallas, TX, USA). LY294002 and Cell counting kit-8 kit were obtained from MedChemExpress (Monmouth Junction, NJ, USA). The following chemicals: 2′,7′-dichlorodihydrofluorescein diacetate (H_2_DCF-DA), 2,2-diphenyl-1-picrylhydrazyl (DPPH), JC-1, and Hoechst 33342 were procured from Thermo Fisher Scientific (Waltham, MA, USA). An apoptosis detection kit (Annexin V–FITC) was obtained from ENZO Life Sciences (Farmingdale, NY, USA).

### 2.3 ABTS Scavenging Assay

The antioxidant activity of OPWE was assessed using an ABTS assay, following previously established methodologies.^5^ ABTS, peroxidase, and H_2_O_2_ were mixed in appropriate proportions to generate an ABTS^+^ solution. Test samples were reacted with freshly prepared ABTS^+^ solution for 5 min. The absorbance was determined spectrophotometrically at 410 nm. Vitamin C served as a calibration curve. All experiments were conducted in triplicate.

### 2.4 DPPH Free Radical Scavenging Assay

DPPH assays were performed according to the method of Tsou et al.^5^ Under dark conditions, DPPH was dissolved in methanol, and a 200 mM stock solution was prepared. Samples were reacted with DPPH for 30 min, and then the absorbance was determined at 517 nm. Catechin as a calibration. All experiments were repeated in triplicate.

### 2.5 Total Polyphenol Content

The total polyphenol content of OPWE was determined according to previously established methods, using gallic acid as a standard curve.^5^ Test samples were reacted with Folin–Ciocalteu reagent (0.2 N) and 10% Na_2_CO_3_ solution for 20 min, and then the absorbance was determined at 700 nm. All experiments were conducted in triplicate.

### 2.6 Total Flavonoid Content

The total flavonoid content of OPWE was ascertained according to established methods.^5^ Total flavonoid content was determined by sequentially adding 5% NaNO_2_, 0.1% AlCl_3_, 1 M NaOH, and deionized water. After reaction, the samples were determined at 510 nm, using catechins as a calibration. All experiments were repeated three times.

### 2.7 Characterization of OPWE Components

The chemical constituents of OPWE was determined by high-performance liquid chromatography (HPLC) and liquid chromatography–mass spectrometry (LC-MS), according to the procedure reported by Tsou et al.^5^ A C18 column was used for separation under gradient elution, with a mobile phase of water and acetonitrile-containing additives. The detection wavelength was set at 200–400 nm, generating a 3D chromatogram for preliminary component identification. LC-MS analysis, performed using electrospray ionization in positive and negative modes, enabled structural identification of OPWE components based on mass spectrometry data, confirming the presence of key bioactive compounds.

### 2.8 Cell Culture and Treatment

Human RPE cell line ARPE-19 was cultured in Dulbecco’s modified Eagle’s medium (Gibco, Thermo Fisher Scientific, Waltham, MA, USA) added with 10% fetal bovine serum (FBS) and antibiotics (Gibco). Plated cells (2 × 10^5^ cells/mL) in 12-well plates and treated with OPWE, hesperidin, or hesperetin for 1.5 h after full adhesion, and subsequently exposed to NaIO_3_ (6 mM) for 18 or 24 h.

### 2.9 Assessment of Cell Morphology and Viability

After treatment, cell morphology was observed under an optical microscope. Cell viability was evaluated using the cell counting kit-8 (CCK-8). After adding 10 μL of reagent to each well, the cells were incubated for 30 min, and absorbance was read at 450–595 nm. Absorbance values were normalized to 100% based on a control group (mock) and presented as percentage values in a bar graph.

### 2.10 Measurement of Apoptotic Cell Populations

After treatment, cells were collected with 0.25% trypsin–EDTA and transferred to a tube. Cells were then stained with Annexin V (0.25 μg/mL) and propidium iodide (1 μg/mL) for 1 h, and green (FITC) and red (PE) fluorescence were measured by flow cytometry. Apoptotic cell populations were quantified as the sum of Q2 (early apoptosis) and Q4 (late apoptosis).

### 2.11 Detection of Intracellular ROS Accumulation

For ROS analysis, the cells were incubated for 1 h with 2 μM H_2_DCF-DA. The dye was then removed, and the cells were collected with 0.25% trypsin-EDTA. Fluorescence signals were detected using the flow cytometer, measuring green fluorescence (FITC). Statistical values were normalized to 100% based on the control group (mock) and presented as percentage values in a bar graph.

### 2.12 Detection of Mitochondrial Damage

To assess mitochondrial damage, cells were incubated with JC-1 dye (2 μg/mL) for 50 min. Afterward, Hoechst 33342 (1 μg/mL), a DNA-specific fluorescent dye, was introduced and incubation continued for an additional 10 min. Fluorescence signals were detected using an inverted fluorescence microscope, and both green and red fluorescence signals were recorded. Statistical values were calculated as the ratio of red fluorescence to green fluorescence.

### 2.13 Western Blotting

Treated cells were lysed with RIPA buffer to obtain total protein. The extracted proteins were separated by 10% SDS-PAGE electrophoresis and transferred to a PVDF membrane. The membrane was blocked with 5% skim milk, then it was incubated overnight with a primary antibody (Santa Cruz: PI3K p110, Bax, β-actin, GAPDH; ABclonal: p-Akt, HIF-1α, BNIP3, cytochrome C, cleaved caspase-3, cleaved PARP), followed by an HRP-conjugated secondary antibody. Protein bands were detected using ECL reagent and visualized using an imaging system (MultiGel-21 imaging system, Top-Bio, New Taipei City, Taiwan). Quantification was performed using EvolutionCapt software (Vilber Lourmat, Marne-la-Vallee, France). The expression levels of target proteins were normalized to β-actin or GAPDH (loading controls).

### 2.14 Animal Model

C57BL/6 mice (8-week-old) were purchased from the National Laboratory Animal Center (Taipei, Taiwan). All animal experimental procedures were approved by the Institutional Animal Care and Use Committee of Chung Shan Medical University (approval No. 2595). Mice were housed in standard cages under a 12 h light-dark cycle and randomly divided into three groups (six mice per group): mock, NaIO_3_, and NaIO_3_ + OPWE. Before NaIO_3_ treatment, the NaIO_3_ + OPWE group received oral OPWE (1 g/kg) for 7 days. On the seventh day, both the NaIO_3_ and NaIO_3_ + OPWE groups were injected (tail vein injection) with NaIO_3_ (40 mg/kg). The NaIO_3_ + OPWE group continued to receive oral OPWE for the subsequent 7 days. On the seventh day after the NaIO_3_ injection, retinal function was assessed through ERG. Mice were euthanized, and ocular tissues were obtained for hematoxylin and eosin (H&E) staining.

### 2.15 Histological Analysis and Retinal Thickness Measurement

After enucleation, the mice were fixed with 10% formalin, embedded in paraffin, sectioned, and stained with H&E for retinal histological analysis. Retinal thickness, including the total retina, outer nuclear layer (ONL), and inner nuclear layer (INL), was quantified at 100 μm intervals from the nasal to the temporal side according to previously described methods,^5^ and the results were graphically represented.

### 2.16 Electroretinography (ERG) Analysis

Electroretinography (ERG) testing was performed on day 7 after NaIO_3_ injection. Before testing, mice were kept in complete darkness for 12 h, and their pupils were dilated with 0.5% tropicamide and 0.5% phenylephrine hydrochloride (Santen Pharmaceutical, Osaka, Japan). Under general anesthesia, the pupils were aligned with the lens of a Ganzfeld ERG system (Phoenix Research Laboratories, Pleasanton, CA, USA). Retinal function was assessed using flash stimuli of varying intensities (−2, −1, 0, 1, 2, and 3 cd·s/m^2^ based on the operating manual. The a-wave amplitude was measured as the change from baseline to trough; b-wave amplitude was measured as the change from the trough of the a-wave to the peak of the b-wave.

### 2.17 Statistical Analysis

Both *in vitro* and *in vivo* data were tested for normality using the Shapiro–Wilk test and for homogeneity of variance using Levene’s test to confirm suitability for parametric statistical methods. Differences between groups were analyzed using one-way ANOVA with Tukey’s post hoc test; *p* < 0.05 was considered statistically significant. Data are presented as mean ± standard deviation (SD). In statistical graphs, notable differences between groups are denoted by different letters; no notable differences are indicated by the same letters. *In vivo* analysis, “*” indicating a notable difference between the control (mock) group and the NaIO_3_ group (*p* < 0.05), and “#”for notable differences between the NaIO_3_ + OPWE and NaIO_3_ groups (*p* < 0.05).

## 3 Results

### 3.1 Evaluation of Antioxidant Potential, Polyphenol, and Flavonoid Content of OPWE

Higher levels of polyphenols and flavonoids in plant products indicate stronger biological properties, such as antioxidant and anti-inflammatory properties.^36^ Flavonoids have a variety of biological effects, including inhibition of key enzymes in mitochondrial respiration; prevention of coronary heart disease; and anti-inflammatory, antitumor, and antibacterial effects.^37^ To enhance extraction efficiency and antioxidant activity, we extracted polyphenols and flavonoids from fresh orange peels through ultrasound-assisted water extraction.^5^ The resultant extract was stored at −80 °C to maintain stability. Antioxidant properties were evaluated based on DPPH free radical scavenging activity, ABTS^+^ antioxidant capacity, total polyphenol content, and total flavonoid content; the results are summarized in Table 1. OPWE exhibited strong antioxidant activity, emerging as a potent free radical scavenger. It exerted scavenging effects on DPPH (2.58 ± 0.01 μmol of catechin/g) and ABTS^+^ (130.11 ± 0.15 μmol of vitamin C equivalent/g). The total polyphenol and total flavonoid content of OPWE were 14.11 ± 0.01 μmol of gallic acid/g and 1.49 ± 0.01 nmol of catechin, respectively. Notably, 3D high-performance liquid chromatography (Figure 1) and LC-MS identified at least 28 active components (Table 2), including hesperidin and hesperetin, two well-known compounds in orange peels. These findings support the potential therapeutic properties of OPWE and suggest that orange peel polyphenols and flavonoids hold promise as valuable antioxidant compounds.

**Table 1:** Antioxidant Activity OPWE^a^

Fruit extract	DPPH	ABTS^+^	Polyphenol	Flavonoid
	Scavenging activity (μmol CE/g sample)	Scavenging activity (μmol VCE/g sample)	Content (μmol GAE/g sample)	Content (nmol CE/g sample)
OPWE	2.58 ± 0.01	130.11 ± 0.15	14.11 ± 0.01	1.49 ± 0.01

a OPWE were sonicated at 50 °C and then subjected to antioxidant assays. Free radical scavenging activity was assessed using the DPPH assay; antioxidant content was expressed as Trolox equivalents; total phenolic content was determined using the Folin–Ciocalteu colorimetric method ; and total flavonoid content was determined using the NaNO_2_–AlCl_3_–NaOH method. Data are presented as mean ± SD (*N* = 3). Vitamin C equivalent, VCE; catechin equivalent, CE; gallic acid equivalent, GAE.

**Figure 1:**
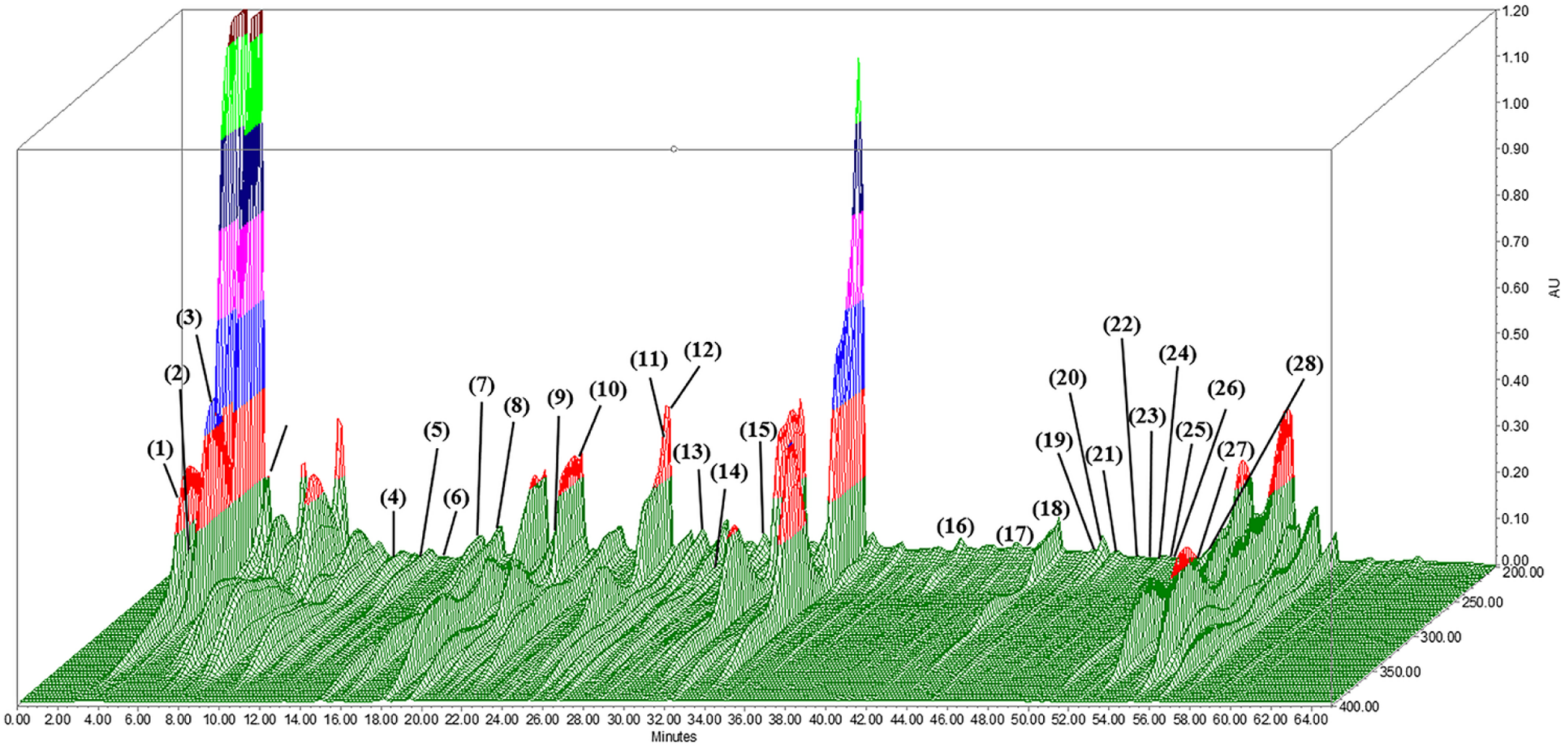
Chromatograms (280 nm) depicting polyphenols and flavonoids in OPWE. The identified compounds and their retention times are listed in Table 2. Data are presented in arbitrary absorption units.

**Table 2:** Composition of Orange Peel Water Extract: Identification of 28 Flavonoids and Phenolic Acids

No.	Compound	Retention time (min)	Formula	References
1	Caffeic acid *O*-glucoside	2.60	C_15_H_18_O_9_	^38^
2	Luteolin	2.82	C_15_H_10_O_6_	^39^
3	Isosakuranetin	2.95	C_16_H_14_O_5_	^40^
4	Quercetin-rutinoside	11.37	C_27_H_30_O_16_	^39^
5	Sinapoyl d-glucoside	12.87	C_17_H_22_O_10_	^42^
6	Apigenin 6,8-di-C-glucoside	13.97	C_27_H_30_O_15_	^42^
7	Stellarin-2	15.15	C_28_H_32_O_16_	^43^
8	Narirutin-4’-glucoside	15.63	C_33_H_42_O_19_	^40^
9	Hesperetin	19.68	C_16_H_14_O_6_	^38,41^
10	Apigenin-O,C-pentosyl-hexoside	20.97	C_26_H_28_O_14_	^42^
11	Eriocitrin	23.68	C_27_H_32_O_15_	^40^
12	Isovitexin-2”-O-glucoside	24.43	C_27_H_30_O_15_	^42^
13	Narirutin	26.27	C_27_H_32_O_14_	^40,41^
14	Diosmin	28.15	C_28_H_32_O_15_	^42^
15	Hesperidin	29.35	C_28_H_34_O_15_	^39,40^
16	Poncirin	39.23	C_28_H_34_O_14_	^44^
17	Citrusin III	41.78	C_36_H_53_N_7_O_9_	^44^
18	5-Hydroxy-3′,4′,5′-trimethoxyflavone	44.33	C_18_H_16_O_6_	^42^
19	Naringenin	45.32	C_15_H_12_O_5_	^39,41^
20	Isoinsenestin	46.05	C_20_H_20_O_7_	^45^
21	Gossypetin hexamethyl ether	46.95	C_21_H_22_O_8_	^43^
22	Sinensetin	47.48	C_20_H_20_O_7_	^40,46^
23	Hexamethylquercetagetin	48.35	C_21_H_22_O_8_	^46^
24	Nobiletin	48.80	C_21_H_22_O_8_	^40,46^
25	Tetramethylscutellarein	48.97	C_19_H_18_O_6_	^46,47^
26	3,5,6,7,8,3′,4′-Heptamethoxyflavone	49.57	C_22_H_24_O_9_	^40,46^
27	Tangeretin	50.13	C_20_H_20_O_7_	^40,46^
28	Kaempferol-3-*O*-arabinoside	51.87	C_20_H_18_O_10_	^41^

### 3.2 OPWE Suppresses NaIO_3_-Induced Apoptosis in ARPE-19 Cells

The cytotoxicity of OPWE in ARPE-19 cells was first assessed after treatment for 24 h. As shown in Figure 2A, OPWE did not significantly affect cell viability even at the maximum tested dose (40 mg/mL), indicating the absence of cytotoxic effects. To assess the protective effect of OPWE, ARPE-19 cells were pretreated with various doses (0, 1.25, 2.5, 5, and 10 mg/mL) of OPWE and cotreated with NaIO_3_ for 24 h. OPWE significantly suppressed NaIO_3_-induced cell death in a dose-responsive manner, conferring the most pronounced protection at 5 and 10 mg/mL concentrations. Therefore, the 5 mg/mL concentration was selected as the highest dose for subsequent experiments (Figure 2B).

**Figure 2:**
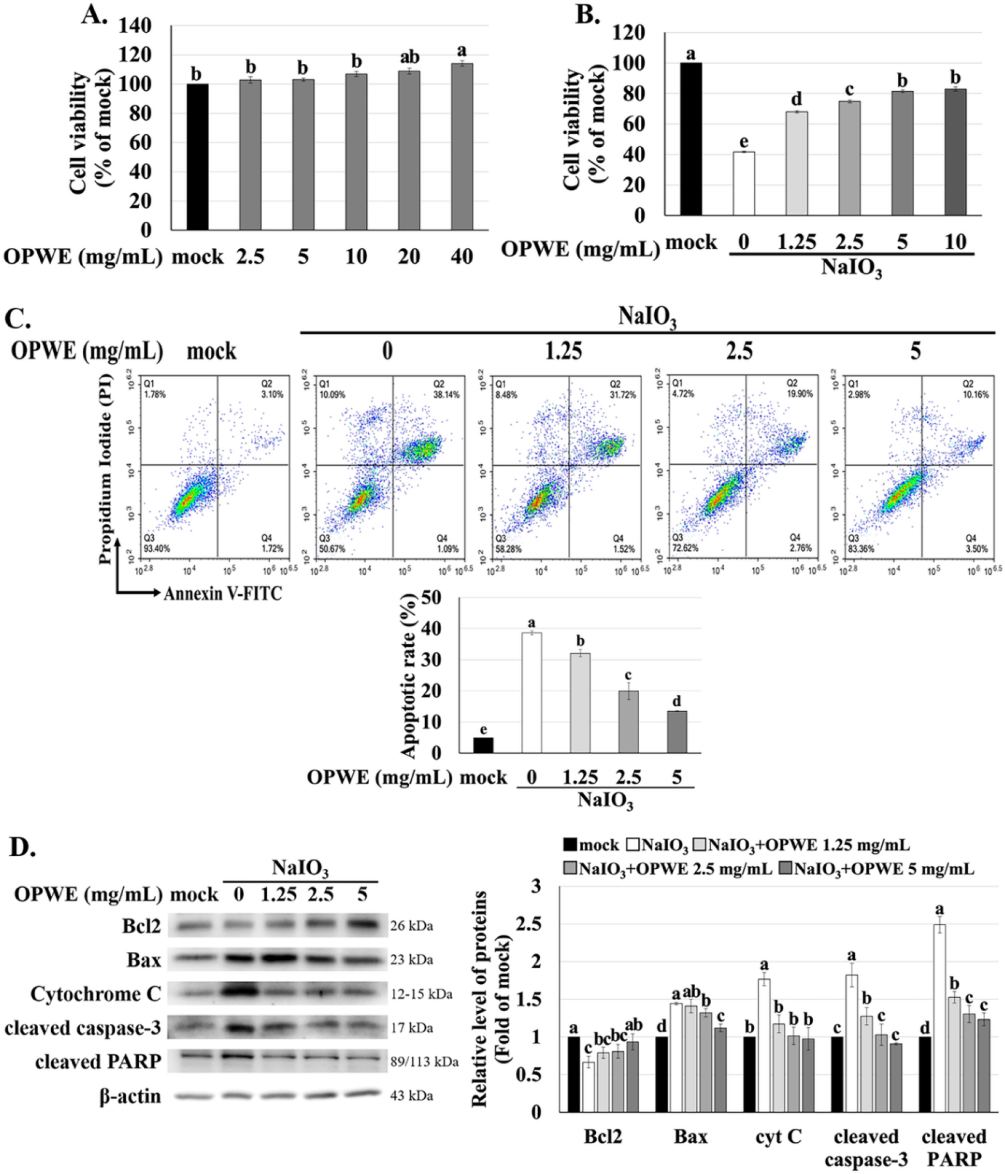
Effects of OPWE and NaIO_3_ on cell viability and apoptosis in ARPE-19 cells. (A) Cell viability after 24 h treatment with varying doses of OPWE. (B) The cells pretreated with OPWE (1.5 h) and exposed to NaIO_3_ (24 h) were assessed for cell viability by CCK-8 assay. (C) Flow cytometry was used to measure cell apoptosis. (D) Western blot analysis was used to analyze the expression of apoptosis-related proteins, with the mock group serving as the standard. Data are presented as mean ± SD (*n* = 3). Different letters (a–e) indicate significant differences within each group (*p* < 0.05).

Research indicates that NaIO_3_ induces RPE cell death through both caspase-3/7/8-dependent apoptosis and caspase-independent necroptosis.^10,48^ To investigate whether OPWE suppresses NaIO_3_-induced apoptosis, the extent of early plus late apoptosis (Q2 + Q4) was assessed using annexin V-FITC/propidium iodide staining.^49^ NaIO_3_ significantly increased the overall apoptosis rate by approximately 7-fold compared with the mock group (*p* < 0.05). Pretreatment with OPWE reduced both early and late apoptosis in NaIO_3_-treated cells (Figure 2C).

We subsequently performed Western blotting to analyze the expression levels of apoptosis-related proteins. OPWE reduced the NaIO_3_-induced upregulation of Bax, a proapoptotic protein, while increasing the expression of Bcl-2, an antiapoptotic protein. In addition, OPWE inhibited the activation of cytochrome c, cleaved caspase-3, and cleaved PARP (Figure 2D), all of which are key mediators of mitochondrial-dependent apoptosis. These findings confirm that OPWE suppresses NaIO_3_-induced apoptosis in ARPE-19 cells by regulating key apoptotic pathways, consistent with the literature.^48,50^

### 3.3 OPWE Mitigates NaIO_3_-Induced Mitochondrial Dysfunction and Oxidative Stress in ARPE-19 Cells

Mitochondrial dysfunction and ROS accumulation, which lead to increased oxidative stress in RPE cells, are closely associated with the pathogenesis of AMD.^4^ Accordingly, we evaluated mitochondrial membrane potential (MMP; through JC-1 staining), and intracellular ROS levels (through H_2_DCF-DA staining). NaIO_3_ increased the intracellular ROS level by approximately 2.2-fold. However, pretreatment with OPWE markedly prevented NaIO_3_-induced ROS production (Figure 3A). Because intracellular ROS accumulation disrupts mitochondrial function and activates the mitochondria-dependent intrinsic apoptotic pathway,^51^ we evaluated MMP as an indicator of mitochondrial integrity. NaIO_3_ considerably reduced MMP in ARPE-19 cells to 35% of the mock group. Pretreatment with OPWE effectively prevented the NaIO_3_-induced reduction in MMP (Figure 3B). These results suggest that OPWE inhibits NaIO_3_-induced ROS-mediated mitochondrial dysfunction, thereby suppressing apoptosis.

**Figure 3:**
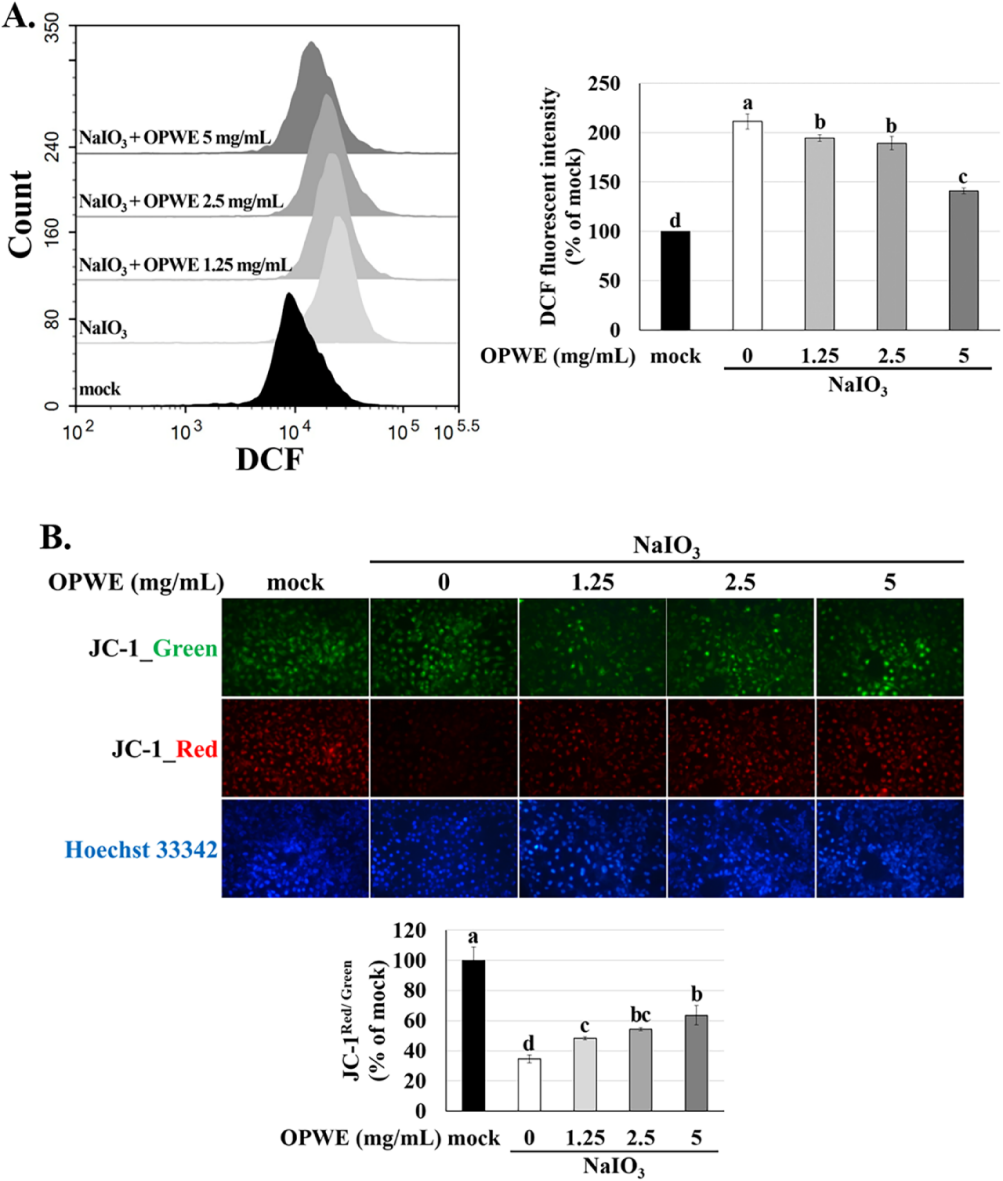
Effects of OPWE and NaIO_3_ on MMP and intracellular ROS levels in ARPE-19 cells. (A) Intracellular ROS levels were evaluated using H_2_DCF-DA staining and flow cytometry. (B) MMP was assessed using JC-1 staining and fluorescence microscopy. Data are presented as the means ± SD (*n* = 3). Different letters (a–d) denote significant differences within each group (*p* < 0.05).

### 3.4 OPWE Modulates PI3K/Akt/HIF-1α/BNIP3 Signaling to Protect ARPE-19 Cells from NaIO_3_-Induced Cytotoxicity

Wang et al. indicate that melatonin protects ARPE-19 cells from NaIO_3_-induced mitochondrial dysfunction and apoptosis by suppressing ROS-driven activation of the HIF-1α/BNIP3-LC3B mitophagy signaling pathway.^21^ We observed similar trends: NaIO_3_ treatment markedly heightened the expression of HIF-1α and BNIP3, while OPWE pretreatment effectively inhibited this phenomenon (Figure 4A). The PI3K/Akt pathway regulates various physiological functions and serves as a key survival pathway by regulating antioxidant defense mechanisms.^11,14,52^ Dysregulation of this pathway has been involved in multiple conditions, such as cancer, diabetes, cardiovascular diseases, and neurological disorders. We further investigated how OPWE regulates NaIO_3_-induced ROS-mediated mitochondrial dysfunction and apoptosis. NaIO_3_ induced the upregulation of PI3K and p-Akt, while OPWE pretreatment attenuated this effect in a dose-dependent manner. (Figure 4B).

**Figure 4:**
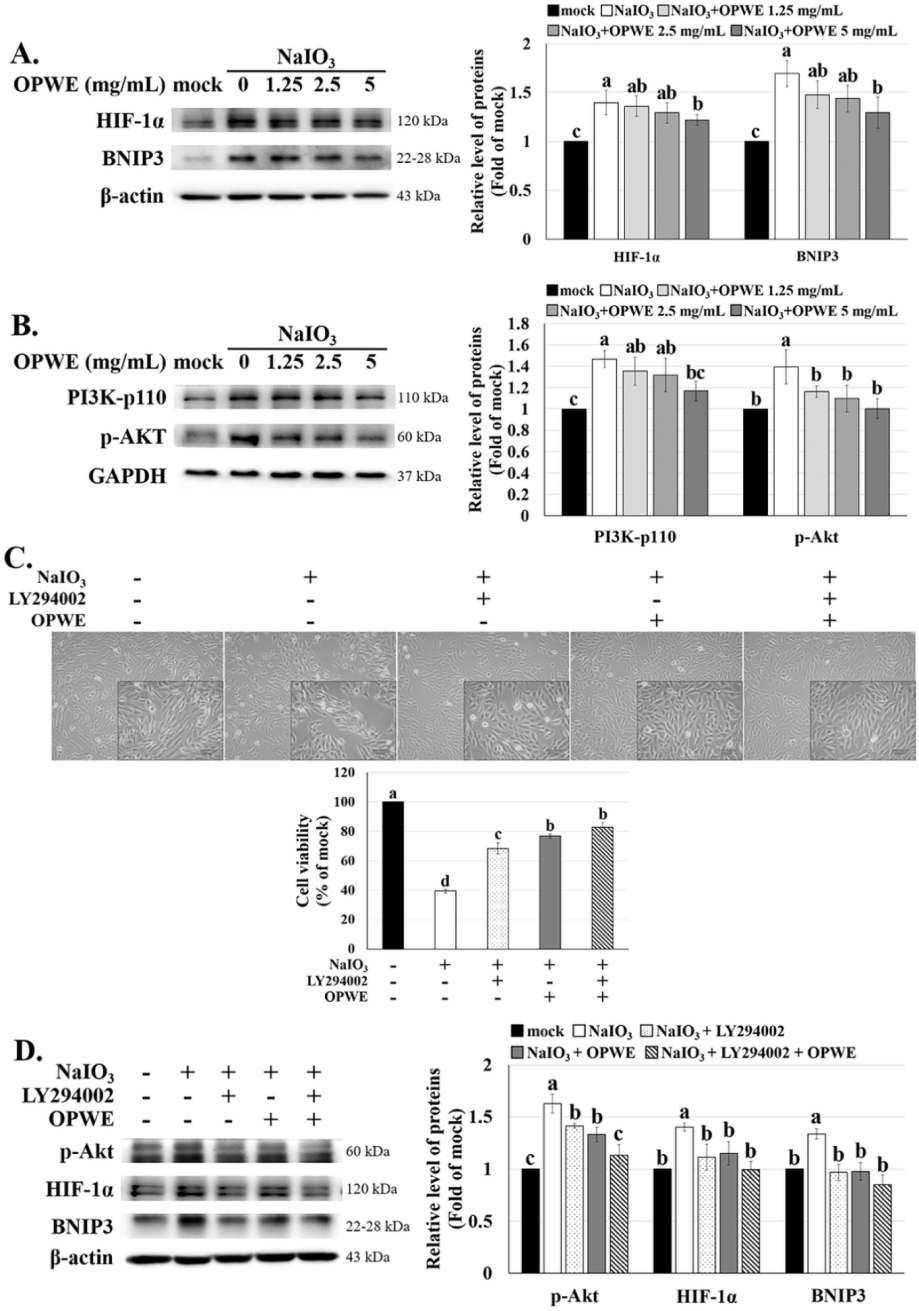
Effects of OPWE and LY294002 on PI3K/Akt signaling, HIF-1α expression, and cell viability in ARPE-19 cells.Western blotting analysis of (A) HIF-1α and BNIP3, and (B) PI3K-p110 and p-Akt levels, with GAPDH or β-actin as controls. (C) ARPE19 cells were pretreated with LY294002 and OPWE for 1.5 h and then treated with NaIO_3_ for 24 h. The cell morphology and cell viability were assessed by microscopy and CCK-8 assay. (D) Expression of p-Akt, HIF-1α, and BNIP3 was normalized to an internal control. Data are mean ± SD (*n* = 3). Different letters (a–d) indicate significant differences within the groups (*p* < 0.05).

To investigate the role of the PI3K/Akt pathway in NaIO_3_-induced ARPE-19 cytotoxicity, we treated cells with the PI3K inhibitor (LY294002), OPWE, or a combination of both for 1.5 h, and then exposed them to NaIO_3_ for 24 h. LY294002 significantly attenuated NaIO_3_-induced cell death. The combination of LY294002 and OPWE further enhanced cell viability (Figure 4C), supporting the involvement of PI3K/Akt signaling in this cytotoxic process. Moreover, LY294002 significantly inhibited the expression of p-Akt, HIF-1α, and BNIP3 (Figure 4D). According to the above experimental results suggest that OPWE mitigates NaIO_3_-induced ROS-mediated mitochondrial dysfunction and apoptosis by modulating the PI3K/Akt/HIF-1α/BNIP3 pathway. Therefore, PI3K/Akt/HIF-1α/BNIP3 signaling may mediate the cytoprotective activity of OPWE against NaIO_3_-induced apoptosis in ARPE-19 cells.

### 3.5 Hesperetin Mitigates NaIO_3_-Induced Mitochondrial Dysfunction and Oxidative Stress in ARPE-19 Cells

Since this study aims to explore the AMD model, if the drug is to be effective, it must be able to penetrate the blood-brain barrier (BBB) and the blood-retinal barrier (BRB). Hence, among the 28 active components of OPWE, we will give priority to the ingredients that can penetrate the BBB. Previous studies have shown that citrus flavanones, including hesperidin, hesperetin, and neohesperidin, are known to have antioxidant activity and can penetrate the BBB.^53^ Therefore, this study will utilize hesperidin and hesperetin, which are contained in the extract, for subsequent experiments. Compared with cells treated with NaIO_3_, hesperidin and hesperetin significantly increased cell survival rate and decreased ROS generation, but hesperetin had a better protective effect than hesperidin and exerted a similar protective effect as OPWE (Figure 5). These findings indicate hesperetin as a key bioactive component in OPWE.

**Figure 5:**
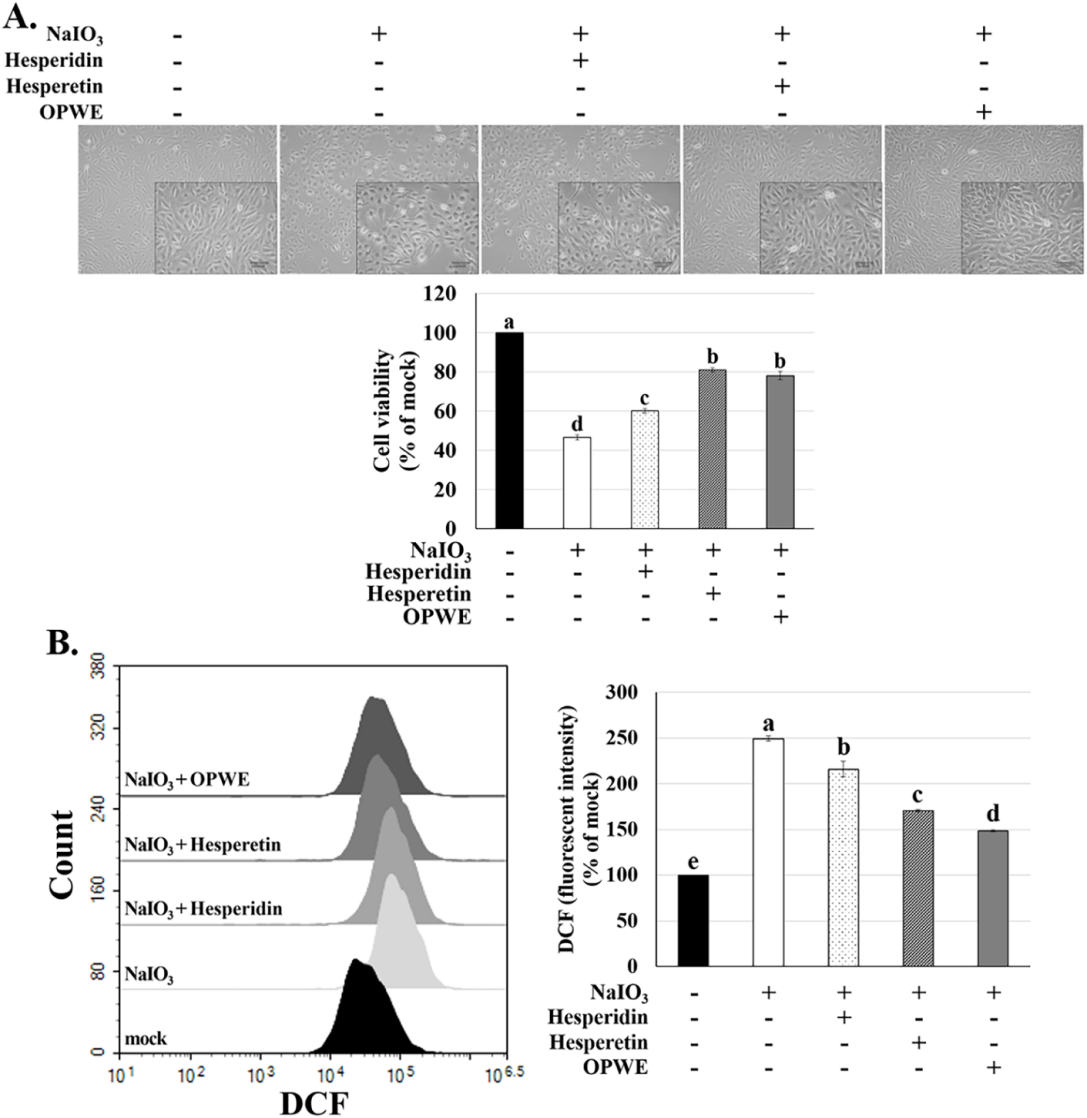
Effects of hesperidin, hesperetin, and OPWE on cell viability and intracellular ROS levels in ARPE-19 cells. ARPE-19 cells were first treated with hesperidin (100 μM), hesperetin (100 μM), or OPWE (5 mg/mL) for 1.5 h and then with NaIO_3_ for 24 h. (A) Cell morphology under a microscope. (B) Cell viability after different treatments was measured using a cell counting kit-8 assay. (C) Intracellular ROS levels following treatment with hesperidin, hesperetin, or OPWE and NaIO_3_ were assessed using H_2_DCF-DA staining and flow cytometry. Data are presented as the means ± SD (*n* = 3). Different letters (a–e) indicate significant differences (*p* < 0.05) within the groups.

To determine whether hesperetin prevents NaIO_3_-induced cell death by mitigating mitochondrial dysfunction and oxidative stress in ARPE-19 cells, we evaluated intracellular ROS levels and MMP. Hesperetin significantly reduces NaIO_3_-induced ROS accumulation (Figure 6A) and mitigates MMP imbalance (Figure 6B). According to the above results, hesperetin can protect ARPE-19 cells against NaIO_3_-induced oxidative stress and mitochondrial dysfunction.

**Figure 6:**
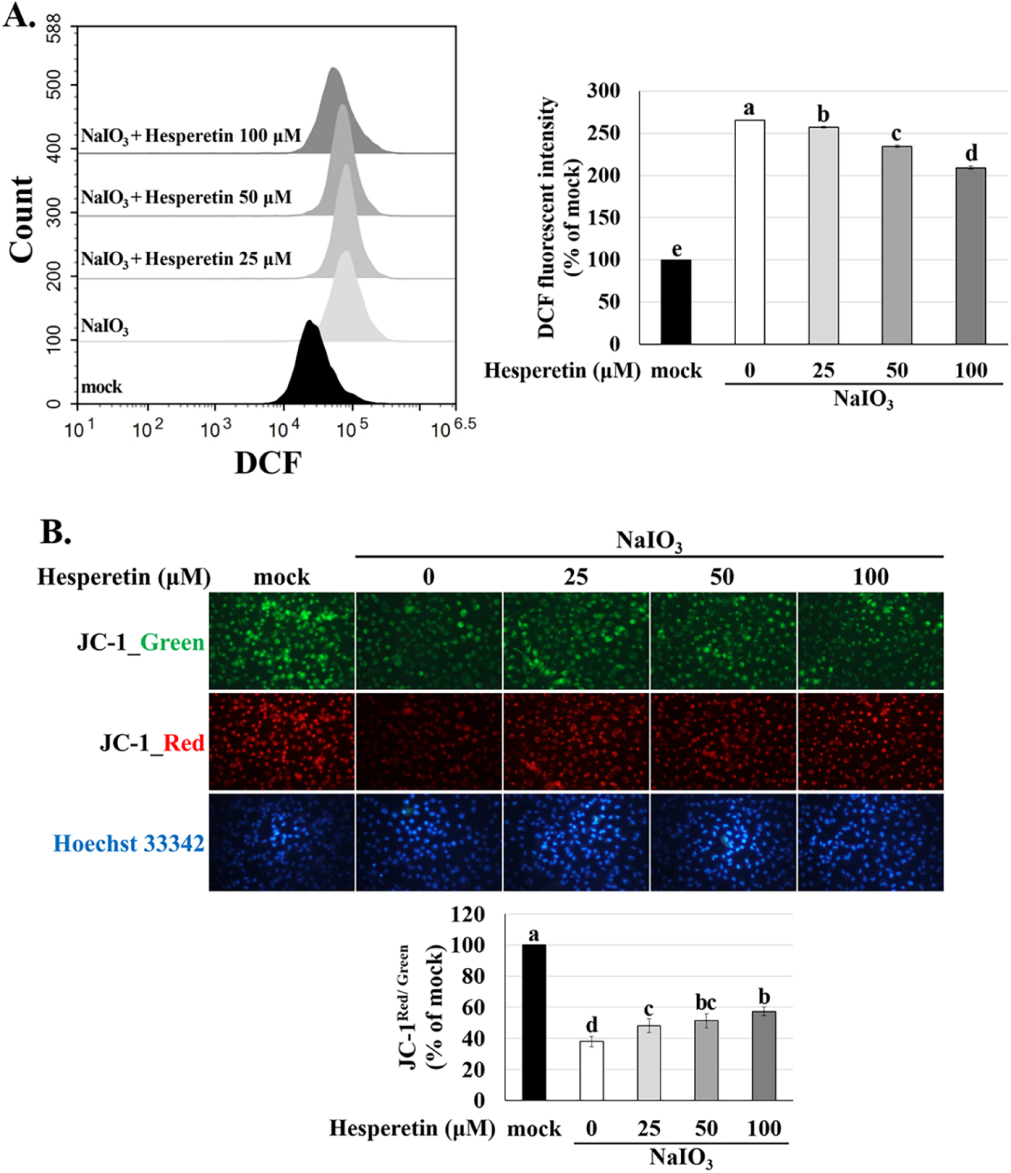
Effects of hesperetin and NaIO_3_ on intracellular ROS levels and MMP in ARPE-19 cells. ARPE-19 cells were first treated with hesperetin for 1.5 h and then with NaIO_3_ for 24 h. (A) Intracellular ROS levels were evaluated using H_2_DCF-DA staining and flow cytometry. (B) MMP was assessed using JC-1 staining and fluorescence microscopy. Data are presented as the means ± SD (*n* = 3). Different letters (a–e) in the statistical graphs indicate significant differences (*p* < 0.05) within each group.

### 3.6 OPWE Ameliorates NaIO_3_-Induced Retinal Structural and Functional Impairments in C57BL/6 Mice

To investigate the protective effects of OPWE on NaIO_3_-induced retinal dysfunction *in vivo*, mice were administered OPWE (1 g/kg) via oral gavage for 7 consecutive days, then subjected to an injection 40 mg/kg of NaIO_3_ (intravenous injection, IV).^5,54^ Thereafter, OPWE was administered daily through oral gavage until the mice were euthanized on the 14th day. Evidence suggests that NaIO_3_-induced retinal damage markedly reduces photoreceptor activity (measured in terms of the a-wave amplitude in ERG) and bipolar cell function (measured in terms of the b-wave amplitude).^55,56^ Retinal function was evaluated using ERG before euthanizing the mice. The results revealed that the a-wave and b-wave amplitudes were significantly lower in the NaIO_3_ group (35.77 ± 2.95 and 88.8 ± 7.96 μV, respectively; *p* < 0.05) than in the mock group. Furthermore, the a-wave and b-wave amplitudes were significantly higher in the OPWE + NaIO_3_ group (158.28 ± 53.63 and 237.48 ± 42.81 μV, respectively; *p* < 0.05) than in the NaIO_3_ group. These results indicate that OPWE restored retinal function toward normal levels (Figure 7A–C).

**Figure 7:**
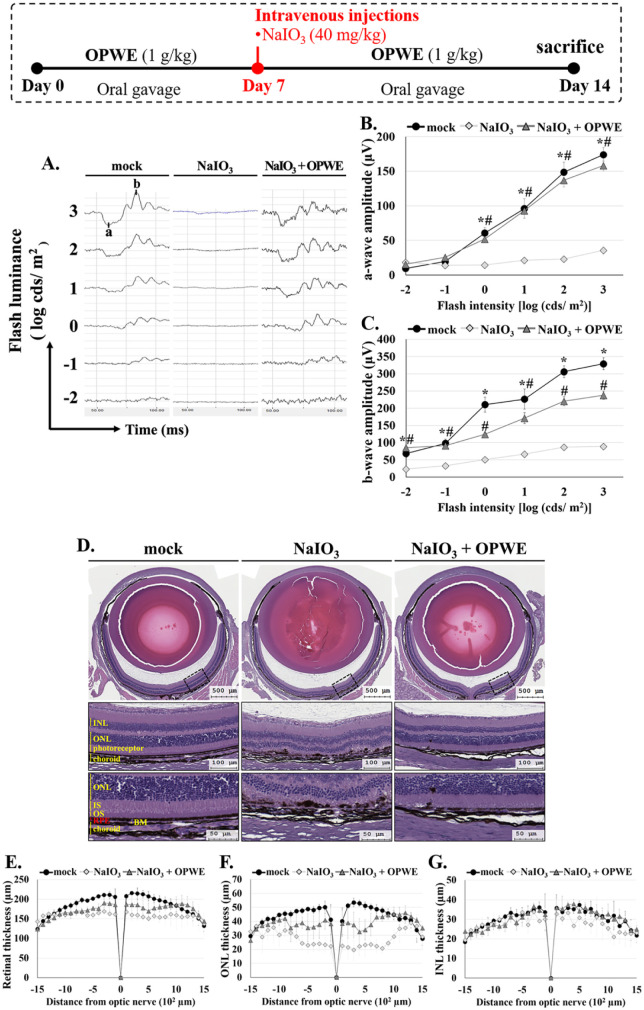
Protective effects of OPWE against NaIO_3_-induced retinal damage in C57BL/6 mice. (A) Retinal electrical responses were evaluated using ERG 7 days after NaIO_3_ treatment. Scotopic ERG responses were recorded at various flash intensities (log cd/m^2^). (B) Average a-wave amplitude and (C) b-wave amplitude plotted as functions of ERG flash intensity. (D) Retinal morphology (H&E staining) 7 days after NaIO_3_ treatment. (E–G) Statistical graphs depict the measured thicknesses of the total retina, outer nuclear layer (ONL), and inner nuclear layer (INL). Data are presented as mean ± standard deviation values (*n* = 4). * indicates a significant difference (*p* < 0.05) between the control group (mock) and NaIO_3_; # indicates a significant difference (*p* < 0.05) between NaIO_3_ + OPWE and NaIO_3_.

To investigate the protective effects of OPWE on retinal structure, we measured the thickness of the total retina, ONL, and INL. H&E staining revealed that NaIO_3_ treatment led to retinal thinning, irregular outer segments, and RPE layer damage, accompanied by disorganization of the inner and outer segments of the photoreceptors (Figure 7D–G). Furthermore, ONL and INL thicknesses were more reduced in the NaIO_3_ group than in the mock group. However, OPWE treatment markedly increased the thickness of the ONL, INL, and total retina, restoring these parameters toward normal levels.

## 4 Discussion

Citrus fruits are vital economic crops, with particular importance in the food processing industry, where they are widely used in juice production. More than 100 million tons of citrus fruits are processed annually, with sweet orange being the most commonly used variety.^57,58^ Approximately 20% of the fruit weight becomes byproducts such as peels, pulp, and seeds, which are often classified as agri-food waste. These byproducts can negatively affect the environment if not managed appropriately. Sweet orange byproducts, particularly peels, are rich in bioactive phenolic compounds, such as flavonoids and phenolic acids.^57,58^ These compounds have been investigated for applications in skincare,^58^ antiaging,^59^ anticancer,^60^ and neuroprotective agents.^35^ Their potential role in retinal health remains largely unexplored. Orange peel flavonoids have been demonstrated to exert antioxidant effects, mitigating lipid peroxidation and oxidative stress in older adults.^61^ Molan et al. attributed the antioxidant properties of citrus peels and seeds to their phenolic compounds.^62^ Chen et al. reported that pretreatment with sweet orange peel extract markedly reduced lipid peroxidation and protected against CCl_4_-induced oxidative damage in rats.^63^ Similarly, we found that OPWE exhibited strong antioxidant activity, as evidenced by its potent DPPH and ABTS^+^ free radical scavenging effects. Furthermore, OPWE contained considerable levels of polyphenols and flavonoids. We used 3D-HPLC and LC-MS to identify at least 28 active components, including hesperidin and hesperetin, highlighting the therapeutic potential of OPWE. These findings suggest that OPWE is a valuable source of antioxidants.

The primary flavonoids in citrus plants are hesperidin and hesperetin (typically obtained through hesperidin hydrolysis). Hesperidin and hesperetin exhibit diverse biological activities, such as antioxidant and anti-inflammatory effects,^64,65^ and may protect against neurodegenerative disorders (particularly Alzheimer’s disease), cancer, and cardiovascular diseases.^66–68^ Epidemiological evidence shows that Gopinath et al. reported a significant relationship between increased hesperidin intake and a reduced probability of late-stage AMD.^32^ Additionally, Zhu et al. reported that hesperetin activates the Keap1–Nrf2/HO-1 pathway, enhances the expression of intracellular antioxidants (GSH, SOD, and HO-1), and confers protection to ARPE-19 cells against H_2_O_2_-induced oxidative stress.^69^ In our study, both hesperidin and hesperetin enhanced the survival of NaIO_3_-treated ARPE-19 cells. Hesperetin was 20% more effective than hesperidin. Similar results were observed for the inhibition of ROS accumulation, indicating that hesperetin was superior to hesperidin in terms of efficacy. Evidence suggests that hesperidin has antioxidant properties and can ameliorate traumatic brain injury.^70^ Few studies have used NaIO_3_-treated experimental models to explore whether hesperetin protects against retinal damage. In the present study, hesperetin reduced NaIO_3_-induced intracellular ROS accumulation and mitigated MMP imbalance in a concentration-dependent manner. Previous studies have shown that hesperidin (hesperetin-7-O-rutinoside) is deglycosylated into hesperetin by the action of β-d-glucosidase. Choi et al. reported that hesperetin exhibited stronger radical scavenging activity than hesperidin in antioxidant assays (DPPH and ABTS), and also demonstrated more pronounced anti-inflammatory effects in RAW 264.7 cells, which is consistent with our findings.^33^ Therefore, we suggest that hesperetin is a key bioactive component of OPWE.

NaIO_3_ is a selective oxidant that generates ROS, leading to oxidative stress and severe RPE cell damage, characterized by swelling, necrosis, detachment, and vacuolization. These changes result in photoreceptor degeneration, retinal thinning, and structural disorganization.^10,71^ Our *in vivo* experiments indicated that OPWE alleviated NaIO_3_-induced retinal distortion and thinning, particularly in the ONL and the inner and outer photoreceptor segments. ERG revealed that the a-wave and b-wave amplitudes were significantly higher in the OPWE + NaIO_3_ group than in the NaIO_3_ group, implying that OPWE effectively restored visual function in mice. These findings suggest that OPWE can mitigate NaIO_3_-induced retinal damage.

NaIO_3_-induced ROS accumulation triggers apoptosis, impairing photoreceptor function and choroidal capillaries, ultimately leading to RPE cell damage.^21,54^ Thus, preventing oxidative stress-mediated RPE cell death may protect against AMD. To the best of our knowledge, the present study is the first to investigate whether OPWE suppresses NaIO_3_-induced apoptosis. OPWE (5 mg/mL) increased the viability of NaIO_3_-treated ARPE-19 cells by approximately 1.96-fold (*p* < 0.05) (Figure 2B), and reduced apoptosis by 2.86-fold (Figure 2C). Western blotting showed that NaIO_3_ upregulated the expression of Bax, cytochrome C, cleaved caspase-3, and cleaved PARP (pro-apoptotic proteins), while inhibiting the expression of Bcl2 (antiapoptotic protein). However, pretreated OPWE suppressed the expression of cytochrome C, cleaved PARP, and cleaved caspase-3, while upregulated the expression of Bcl2 (Figure 2D). Research suggests that ROS accumulation in the retina causes oxidative damage in RPE cells, ultimately leading to apoptosis.^48,50,54^ In our study, NaIO_3_-induced intracellular ROS accumulation and mitochondrial dysfunction triggered apoptosis. Nevertheless, pretreatment with OPWE effectively regulated apoptosis pathways, inhibiting ROS accumulation and mitochondrial dysfunction, and thereby suppressing NaIO_3_-induced apoptosis.

PI3K-dependent and PI3K-independent mechanisms mediate stress-induced activation of Akt.^72,73^ The PI3K/Akt pathway has been implicated in oxidative stress-induced mitochondrial dysfunction and apoptosis in ARPE-19 cells.^74^ By knocking out HIF-1α and BNIP3, Wang et al. demonstrated that these proteins regulate NaIO_3_-induced ROS production, leading to mitochondrial dysfunction-mediated apoptosis.^21^ We previously reported that NaIO_3_ activates the PI3K/Akt pathway in a partially ROS-dependent manner, ultimately leading to apoptosis.^11,75^ Nevertheless, the precise mechanisms underlying NaIO_3_-induced ARPE-19 cell death and its inhibition by OPWE remain unclear. The present study indicated that OPWE inhibited NaIO_3_-induced PI3K activation, Akt phosphorylation, and HIF-1α and BNIP3 expression. Above findings were confirmed using the PI3K inhibitor LY294002 to suggest that OPWE suppresses NaIO_3_-induced ROS-mediated apoptosis in ARPE-19 cells by modulating the PI3K/Akt/HIF-1α/BNIP3 pathway.

## 5 Conclusion

Our findings suggest that antioxidant components in OPWE can inhibit NaIO_3_-induced retinal damage. Of these components, hesperetin emerged as the primary active component responsible for the protective effects of OPWE. Using *in vitro* and *in vivo* models of retinal damage, we demonstrated that OPWE prevents ROS accumulation-induced mitochondrial dysfunction, and suppresses NaIO_3_-induced apoptosis in ARPE-19 cells through the PI3K/Akt/HIF-1α/BNIP3 pathway (Figure 8), and mitigates NaIO_3_-induced retinal structural distortion and photoreceptor cell damage in mice. These findings indicate that OPWE possesses antioxidant properties that support eye health and may help prevent age-related diseases such as AMD. Furthermore, OPWE may be used for the development of functional materials aimed at protecting blood-brain barrier organs (e.g., the brain and the eyes) and may support sustainable utilization of agricultural byproducts.

**Figure 8:**
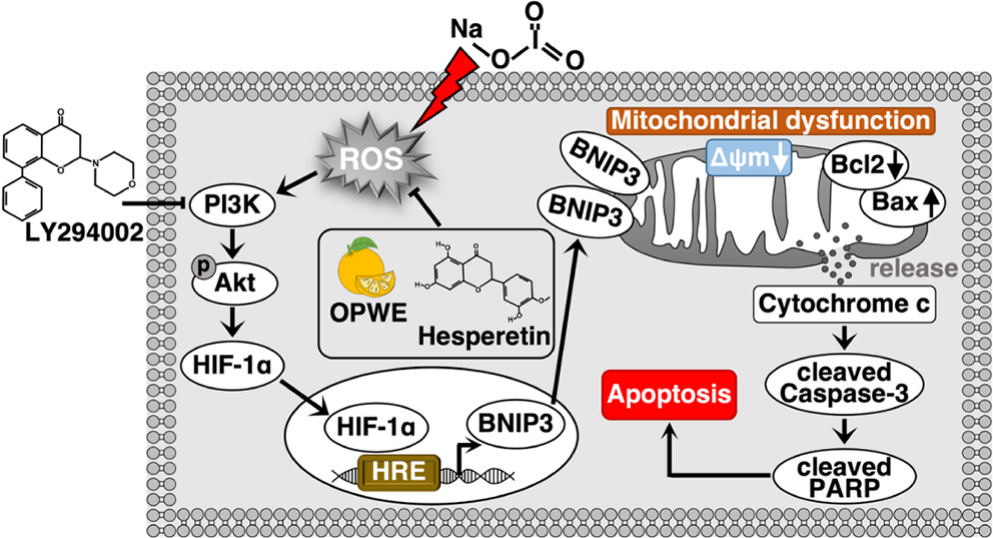
Proposed mechanism through which OPWE protects RPE cells from NaIO_3_-induced apoptosis. OPWE mitigates NaIO_3_-induced oxidative stress by inhibiting the activation of the PI3K/Akt/HIF-1α/BNIP3 pathway, thereby preventing mitochondrial damage and caspase-dependent apoptosis in RPE cells. Thus, OPWE preserves RPE cell integrity, maintaining retinal structure and visual function. Abbreviations: OPWE, orange peel water extract; RPE cell, retinal pigment epithelial cell; NaIO_3_, sodium iodate; HIF-1α, hypoxia-inducible factor-1α; BNIP3, Bcl-2/adenovirus E1B 19 kDa interacting protein 3; PI3K; phosphoinositide 3-kinase; Akt, protein kinase B; PARP, poly­(ADP-ribose) polymerase.

## Data Availability

Data is contained within the article.
